# Pit picking vs. Limberg flap vs. primary open method to treat pilonidal sinus disease – A cohort of 327 consecutive patients

**DOI:** 10.1515/iss-2021-0041

**Published:** 2022-06-27

**Authors:** Dietrich Doll, Sven Petersen, Octavia Alexandra Andreae, Hanne Matner, Henning Albrecht, Lukas E. Brügger, Markus M. Luedi, Gero Puhl

**Affiliations:** Department of Procto-Surgery, St. Marienhospital Vechta, Academic Teaching Hospital of the MHH Hannover, Vechta, Germany; Pilonidal Research Group, Vechta, Germany; Department of General and Visceral Surgery, Asklepios Klinikum Hamburg-Altona, Hamburg, Germany; Department of Visceral Surgery and Medicine, Inselspital, Bern University Hospital, University of Bern, Bern, Switzerland; Department of Anaesthesiology and Pain Medicine, Inselspital, Bern University Hospital, University of Bern, Bern, Switzerland

**Keywords:** Limberg flap, long-term recurrence rate, minimally invasive therapy, pilonidal sinus, pit picking

## Abstract

**Background:**

Minimally invasive methods in pilonidal sinus disease (PSD) surgery are becoming standard. Although long-term results are available for some techniques, long-term outcome data of patients after pit picking is lacking. We aimed at investigating perioperative and long-term outcomes of patients undergoing pit picking, Limberg flap or primary open surgery to treat PSD.

**Methods:**

In a single-centre observational study, we evaluated the outcomes of 327 consecutive patients undergoing PSD surgery between 2011 and 2020.

**Results:**

PSD had recurred in 22% of Limberg flap patients and 62% of pit picking patients at 5 years (p=0.0078; log rank test). Previous pilonidal surgeries, smoking, body mass index, immunodeficiency, and diabetes did not significantly influence the long-term recurrence rate. Primary open treatment was performed for 72% of female patients presenting with primary disease.

**Conclusions:**

Due to its especially dismal long-term results, pit picking should be abandoned, and Limberg flap should be promoted instead, even for primary disease and in females.

## Introduction

A variety of options are available for the treatment of pilonidal sinus disease (PSD). Among them are minimally invasive surgery (such as the so-called “pit picking”) [1], excisional procedures using a lay-open technique, primary closure, and off-midline plastic reconstructive operations. These procedures, which use different types of anaesthesia [2], are associated with advantages as well as disadvantages and may be associated with other cultural or geographical factors [3], [4], [5], [6], [7], [8], [9]. In any case, minimal intervention is recommended, as it is said to be associated with short theatre times, cost efficiency, and superior outcome [10, 11].

Traditionally, medicine and surgery have been based on conservative thinking: a new therapy is only adopted when it has proven its superiority compared to the existing, “old” therapies in use. This has changed in recent years because new therapeutic approaches with allegedly better results have been introduced to the community earlier.

Not surprisingly, an increasing number of therapies claim to be minimally invasive. Among them are sinusectomy, lay-open surgery, debridement of tracts, trephination of tracts, phenolisation of tracts, pit picking, endoscopic surgery, and laser coagulation of pilonidal tracts. However, only a minority of these have been studied in depth over an extended period of time [12].

“Old” knowledge, gained in more than 30 years of use, posits that off-midline closures (such as Limberg or Karydakis or other flap procedures) have the lowest recurrence rates over time [13, 14]. We aimed at comparing pit picking, primary open treatment, and the Limberg flap procedure in a single-centre study with data from our PSD cohort.

## Methods

About 534 consecutive PSD patients were operated on between 1.1.2012 and 31.12.2020. The choice of surgical procedure was at the discretion of the consultant surgeon in charge of the surgical list. Limberg flaps were recommended for recurrent disease that was suitable for this strategy (i.e. without major infectious tissues; disease present in the upper third of the intergluteal fold). Pit picking was generally recommended for any primary disease, but could be changed to excision and primary open treatment when larger tract systems were either seen (large or multiple hair nidus) or suspected (multiple paramedian openings).

Flap patients were observed in hospital till the removal of drain(s), and primary open wounds were observed one night for re-bleeding. Most pit picking patients were discharged the same day. A minimum dataset including the number of previous surgical procedures, surgical details, and follow-up was defined. After studying patient documents, we obtained written informed consent from patients willing to participate in this study. This was followed by a structured interview identifying any symptoms or signs of recurrence and determining whether further surgical procedures were needed following discharge from the hospital. If the interviewer was uncertain about any of the information obtained, the patient was invited for an outpatient visit. Of the n=358 patients successfully contacted, all agreed to take part in the study. Twenty-three patients with incision of acute pilonidal disease were excluded as this was not defined as a surgical procedure for PSD. Two surgical re-debridements of non-healing wounds, one Karydakis flap, and five further operations were excluded from the analysis. All patients with primary as well as recurrent pilonidal disease were enrolled. Thus, n=327 patients with either excision and primary open treatment, Limberg flap, or pit picking were available for group analysis.

Patients with both primary and secondary disease were included. Acute inflammation was drained weeks beforehand, giving enough time for the wound to be converted into a chronic wound without retention. Additional measures, such as the use of methylene blue or the placement of a suction drainage, were implemented intraoperatively at the discretion of the surgeon. Details of ethical approval were provided by the responsible registry.

### Statistics

All data documentation, plausibility checks, and pivot table calculations were done using Microsoft Excel 2013 (Microsoft Office, Microsoft Corp., Sunnyvale, CA, USA). Statistical analyses were carried out using the GraphPad Prism Integrated Statistical Package (GraphPad Prism 5.02, GraphPad Software Inc., San Diego, CA, USA). Categorical as well as continuous variables were represented as percentages or frequencies and mean±SD, respectively. Kaplan Meier Survival curves were calculated using data transfer to GraphPad Prism (as above). The survival curves were compared using the Gehan-Breslow-Wilcoxon test. p<0.05 was regarded as significant.

## Results

Of the 327 patients, n=61 were females and n=266 were males, for a male-to-female ratio of 4:1. The overall mean age was 28±8 years, and women were 3 years younger than men on average. The therapies most often performed were excision and primary open wound treatment (n=197; 60.2%), Limberg flap (n=69; 21.1%), and pit picking (n=61 patients; 18.7%) (Table 1).

**Table 1:** Demographics and disease characteristics for the three surgical PSD treatment groups.

Category	Primary open	Pit picking	Limberg flap	Total
Patients [n], %	197 (60.2)	61 (18.7)	69 (21.1)	327 (100)
Men [n], %	153 (57.5)	53 (19.9)	60 (22.6)	266 (100)
Women [n], %	44 (72.1)	8 (13.1)	9 (14.8)	61 (100)
Age at surgery	28.1 years	27.8 years	27.2 years	27.9 years
Primary disease [n], %	158 (71.5)	49 (22.2)	14 (6.3)	221 (100)
Recurrent disease [n], %	39 (36.8)	12 (11.3)	55 (51.9)	106 (100)
Abscess forming [n], %	135 (87.7)	14 (9.1)	5 (3.2)	154 (100)
Chronic disease [n], %	28 (32.2)	35 (40.2)	24 (27.6)	87 (100)
Acute and chronic disease [n], %	34 (39.5)	12 (14)	40 (46.5)	86 (100)
Smoker [n], %	13	0	9	32
Drug abuse [n], %	5	1	2	8
Crohn/Colitis [n], %	1	0	1	22
BMI, kg/m^2^	27.4	24.7	28.2	27.1
Time in hospital, days	1.5	0.4	3.8	1.8
Five-year recurrence rate	19.2%	62.3%	23.3%	
Ten-year recurrence rate	45.1%	Not available	23.3%	

About 71% of women received primary open treatment, whereas Limberg and pit picking followed 15 and 13%, respectively. By contrast, men underwent fewer primary open treatments (58%). Limberg treatments were performed in one fourth (23%) and pit picking in one fifth (20%) of the male patients. Thus, minimally invasive treatment was used more often in men, and primary open treatment accounted for nearly three quarters of all treatments (Table 2).

**Table 2:** Number of treatments applied, broken down by gender.

Women				Men		
Surgery	No recurrence	Recurrence	Total, %	No recurrence	Recurrence	Total, %
Primary open	38	6	44 (72)	136	17	153 (58)
Pit picking	6	2	8 (13)	36	17	53 (20)
Limberg	8	1	9 (15)	57	3	60 (23)
Total	52	9	61 (100)	229	37	266 (100)

### Perioperative results

The analysis of the surgical procedure duration in theatre (Table 3) shows that PSD surgery takes 18±15 min SD on average, which is about the same for excision and primary open treatment, with 14±10 min. A Limberg flap procedure takes 42±10 min, whereas the pit picking procedure needs an average of 7±4 min (Table 3).

**Table 3:** Time in theatre for the three main procedures [in min].

Surgical technique	Number of patients	Mean time in operating room in minutes, SD
Primary open	197	13.7 [9.5]
Pit picking	61	7.1 [42.0]
Limberg	69	42.3 [10.1]
Total	327	17.6 [15.0]

Patients stayed an average of 1.8±2.2 days in hospital, with a range of 0–15 days, depending on the procedure (Table 4). Patients with Limberg flap surgery stayed in the hospital for 3.8±2.3 days, whereas primary open patients were discharged after 1.5±2 days. The shortest stay was associated with pit picking (0.4±0.6 days; Table 4). Age, gender, diabetes mellitus, immune suppression, inflammatory bowel disease, smoking, drug abuse, and body mass index (BMI) had no statistically significant influence on the length of time spent in the hospital (data not shown).

**Table 4:** Time in hospital depending on the surgical procedure (n=327 patients).

Surgical technique	Number of patients	Mean length of stay in days, SD
Primary open	197	1.5 [2.0]
Pit picking	61	0.4 [0.6]
Limberg	69	3.8 [2.3]
Total	327	1.8 [2.2]

We further investigated the allocation of therapy in relation to primary or recurrent disease: Excision and primary open treatment were allocated in 71% of cases to primary disease patients without preceding operation. Pit picking was used in only 22% of patients being treated for the first time, and in 12% of patients with recurrent disease. The Limberg flap procedure was rarely (6%) applied in primary disease. However, half of all pilonidal patients with recurring disease (52%) were operated using the Limberg flap procedure (Table 5).

**Table 5:** Treatment allocation in relation to primary or recurrent disease status.

Previous open treatments	Therapy/Number of patients, %			Total
	Primary open	Pit picking	Limberg	
0	158 (71%)	49 (22%)	14 (6%)	221
1	39 (38%)	12 (12%)	52 (52%)	103
2			2	2
5			1	1
	197 (60%)	61 (19%)	69 (21%)	327 (100%)

### Long-term results

The raw recurrence rate was 11.7% for primary open treatment, 5.8% for Limberg flap, and 31.1% for pit picking.

**Figure 1:**
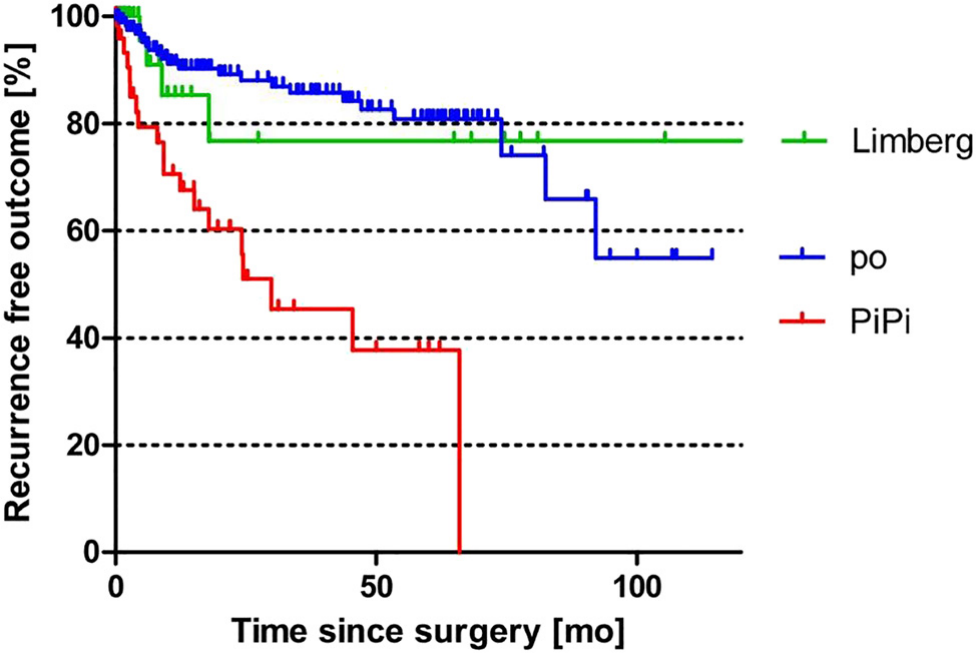
Kaplan-Meier analysis of three surgical pilonidal sinus therapies (po = primary open treatment [n=197 pts.]; Pipi = pit picking [n=61 pts.]; Limberg = Limberg flap [n=69 pts.]).

**Figure 2:**
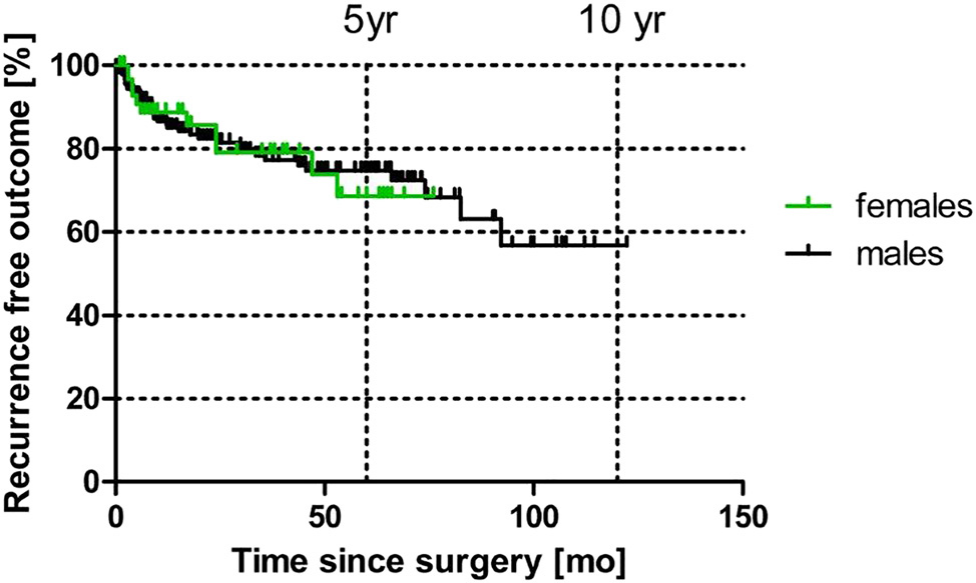
Comparison of gender-specific recurrence rates.

The more accurate time-dependent Kaplan-Meier analysis is shown in Figure 1. Here, the Limberg flap procedure is associated with a recurrence rate of 23% at 10 years. The primary open treatment, by contrast, was shown to have a recurrence rate of 44% at 10 years. For pit picking, only 5-year follow-up results were available, where the recurrence rate was already 60% at this time point. A recurrence-free outcome with pit picking was found to be significantly worse when compared to primary open (p<0.0001; log rank) and Limberg (p=0.0078; log rank test) procedures. These results do not differ when compared for gender differences (Figure 2).

Further, patients with primary PSD (no previous operations; n=221/327 patients) saw the same raw recurrence rate of 32/221 (14%) as that of the 106/327 patients (32%) with previous PSD surgeries, who had a recurrence rate of n=14/106 (13.2%).

**Figure 3:**
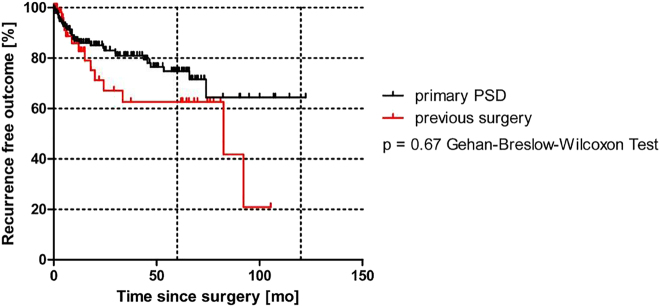
Recurrence-free outcome in primary pilonidal patients vs. patients with previous operations.

Kaplan-Meier analysis underscores these results, as shown in Figure 3 (p=0.67 Gehan-Breslow-Wilcoxon Test).

## Discussion

PSD is a condition affecting both men and women, increasingly common and increasing for unknown reasons. The current analysis is based on data from a large cohort of patients with PSD. These data show that pit picking, a minimally invasive therapy, is easily performed, needs minimal theatre time, and is associated with the shortest hospital stay in our series. Frequently, other authors have highlighted the benefits of this outpatient procedure, which does not require an overnight stay in the hospital [15], [16], [17], [18]. However, its recurrence rate is substandard.

Although Limberg flaps do achieve a low recurrence rate in primary and recurrent disease, pit picking is associated in our cohort with the opposite – a higher recurrence rate.

In our study, the recurrence rate after pit picking was 60% at 5 years after surgery, which equals a 12% recurrence rate per year of follow-up. Considering a large 2018 meta-analysis from Stauffer et al. including 89,583 patients, 6,272 patients who underwent pit picking procedures showed a pit picking recurrence rate of 19% at 5 years of follow-up. This observation is supported by the 23% recurrence rate in Limberg flaps at 10 years, which would be anticipated to be 5%, as published in the world literature [19]. Primary open therapy is associated with a 44% recurrence rate at 10 years, which would be expected to be 19.9% [19]. The recurrence rates in this study exceed the published average, which might be explained by the surgical expertise of our staff. Larger hospitals in cities may act as referral centres, receiving more difficult cases. Thus, negative selection bias may contribute to a higher recurrence rate, as can be seen in our treatment groups.

The recovery after flap treatment includes a few days in the hospital and does not need much more surgical time than an appendectomy. The recurrence rate in flap surgery is the lowest recurrence rate of all pilonidal surgery techniques available to date [20]. Pit picking is faster and linked to a shorter hospital stay, but the high long-term recurrence rate found in our study and reported in the literature does not support the use of this technically easy technique [19].

The advantages of asymmetric open and closed procedures have been proven by evidence over the past three decades [13, 14, 19, 21, 22]. This has been recognized not only for open wound treatment but above all for plastic reconstructive procedures (Karydakis, Limberg) [20, 23]. Although procedures with primary closure are technically more demanding than open techniques [24], they guarantee low complication and recurrence rates [20, 24]. Asymmetric techniques with closure can even be recommended if low-grade infection is present, which is typical for pilonidal disease [25, 26].

The current trend in Australia, Denmark, and other countries [5, 27] is to move away from the primary open techniques toward the so-called minimally invasive techniques. This reflects disinterest in becoming familiar with the flap surgery. However, our data show that it is possible to complete the Limberg flap procedure in an amount of time comparable to that of a hernia repair or laparoscopic cholecystectomy. Thus, longer theatre times are not a reason to avoid flap techniques. Flap recurrence rates are more favourable than those of the other methods applied here [19].

In a 2008 publication, Gips described a remarkable achievement with his newly developed, minimally invasive trephination technique [28]. This procedure is similar to the pit picking method and results in a 22% recurrence rate at 10 years. His study represents the benchmark for a good pilonidal surgical recurrence rate (2% per year of follow-up). Unfortunately, Gips’ results combining low cost and a low long-term recurrence rate have not been replicated by others so far.

The strategy used to estimate the size of the tract system from the number of fistula openings (pori) was not particularly successful in our study. Patients with single porous PSD presented with large subcutaneous tract systems intraoperatively require larger excisions, whereas other patients with multiple openings (>5) revealed only small tracts in the midline. This is in line with the literature saying that there is no robust correlation between the number of skin openings and the size or the extent of the required excision [29]. Thus, the focus on large excisions should be abandoned, and fistula tracts should be “dyed” intraoperatively, followed by meticulous exploration and complete tract excision using trephines or tract excision tools such as the metal-rod-guided AMI FiXcision round knife, which is longer than the trephines available.

Our study has some inherent limitations linked to the retrospective nature of the data, the incorporation of multiple surgeons with multiple levels of technical expertise, and the status as a large urban referral hospital. One-third of our cohort was composed of patients with secondary disease (n=106/327 patients), influencing the choice of treatment. Furthermore, less than one-fifth of our patients with follow-up (n=61/327) were female, undercutting the current ratio of 18/56 in 2017 [30]. Although gender-specific recurrence rates have not been identified so far, there is growing interest in gender-related differences in incidence and recurrence [7, 31, 32].

The results of our study support re-thinking the indications for pilonidal therapy. Although, in other countries, closed asymmetric techniques are increasingly preferred, in our cohort, there is still a high proportion of primary open treatments [27]. This is supported by the fact that 72% of the women in our study chose primary open treatment. This might reflect the dilemma faced by female patients, who avoid asymmetric closure procedures in favour of lay-open techniques due to cosmetic aspects.

However, current guidelines reserve recommendations for primary open treatment for large and extensive PSD only, and generally recommend off-midline closures and flap techniques [18], flaps, and the Karydakis or Limberg technique when aesthetic aspects play a key role.

Pit picking could not be wholeheartedly recommended in recently published national guidelines because the long-term results are still lacking [18]. The results presented here for this technique are not encouraging. Apart from the associated recurrence rate, which exceeds 60% at 5 years due to potentially negative selection (as explained above), pit picking gives the least impressive results, also in comparison with the other methods presented here.

Pit picking could be useful as a technique for the elderly, for outpatients who are averse to surgery, and for patients who have little or no access to social services. Optimized patient selection will be necessary to achieve excellent results like those of Gips [28] in the future.

Based on our current knowledge, indications for primary open treatment—especially in females with primary disease—should be reconsidered, and Limberg flap surgery should be proposed more frequently. As this therapy is already established in many hospitals and accompanied by excellent results, more surgeons should be encouraged to use it. The recurrence rate is low, and complications can be avoided easily [33–35], as demonstrated in a systematic review and network meta-analysis by Bi et al. [20].

## Conclusions

Our study comparing Limberg flap procedures, pit picking, and primary open procedures showed only few advantages for pit picking. Although the technique is minimally invasive, fast, and cheap, it has a recurrence rate of 60%, without a benefit for the majority of patients. We, therefore, recommend the Limberg flap procedure in the treatment of PSD.

## Supplementary Material

Supplementary Material
