# Supplementary material for: Pit picking vs. Limberg flap vs. primary open method to treat pilonidal sinus disease – A cohort of 327 consecutive patients
Source: Innov Surg Sci. 2022 Jun 27;7(1):23–9. doi: 10.1515/iss-2021-0041 (PMC9352183; doi:10.1515/iss-2021-0041)
Supplement: Supplementary file 1 — Supplementary Material [file j_iss-2021-0041_suppl.pdf]

Reviewer Assessment

D. Doll et al.: Pit picking versus Limberg flap versus primary open method to treat Pilonidal Sinus Disease – a cohort of 327 consecutive patients

Reviewers' Comments to Original Submission

Reviewer 1: Mattias Maak

Date received: 07-Feb-2022
Reviewer recommendation: **Return to author for minor modifications**
Reviewer overall scoring: **Excellent**

Assessment Form scores: 5 = High/Yes; 3 = Medium/Adequate; 1 = Low

Is the subject area appropriate for the journal		4			
Does the title clearly reflect the paper's content?	5				
Does the abstract clearly reflect the paper's content	5				
Do the keywords clearly reflect the paper's content?	5				
Does the introduction present the problem clearly?	5				
Are the results/ conclusions justified?		4			
How comprehensive and up-to-date is the subject matter presented?	5				
How adequate is the data presentation?	5				
Are units and terminology used correctly?	5				
Is the number of cases adequate?		4			
Are the experimental methods/ clinical studies adequate?	5				
Is the length appropriate in relation to the content?	5				
Does the reader get new insights from the article?	5				
Please rate the practical significance.	5				
Please rate the accuracy of methods.	5				
Please rate the statistical evaluation and quality control.	5				
Please rate the appropriateness of the figures and tables.	5				
Please rate the appropriateness of the references.	5				
Please evaluate the writing style and use of language.	5				
Please judge the overall scientific quality of the manuscript.	5				
Are the methods used worthy of reproduction in greater deal?	Yes				
Would you be willing to review a revision of this manuscript?	No				

Comments to author: Thank you for this important study.

Since the various minimal invasive treatment options of PSD evolved in rather quick succession over the years, as described in your paper, it is vital for the benefit of our patients to follow up on these techniques in studies like this one.

Of course the number of cases ist rather small over a time period of 9 years, but the data is adequately presented with clear structure and purpose. The data allows and sustains your conclusions.

Just minor changes due to typos should be adressed:
Page 11, Line 57 and following. The text appears to be superscript, also on Page 13 line 7.

Reviewer 2: Dirk Wilhelm

Date received: 07-Feb-2022
Reviewer recommendation: **Return to author for minor modifications**
Reviewer overall scoring: **High**

Assessment Form scores: 5 = High/Yes; 3 = Medium/Adequate; 1 = Low

Is the subject area appropriate for the journal		4			
Does the title clearly reflect the paper's content?	5				
Does the abstract clearly reflect the paper's content		4			
Do the keywords clearly reflect the paper's content?	5				
Does the introduction present the problem clearly?		4			
Are the results/ conclusions justified?		4			
How comprehensive and up-to-date is the subject matter presented?		4			
How adequate is the data presentation?	5				
Are units and terminology used correctly?	5				
Is the number of cases adequate?	5				
Are the experimental methods/ clinical studies adequate?		4			
Is the length appropriate in relation to the content?			3		
Does the reader get new insights from the article?		4			
Please rate the practical significance.		4			
Please rate the accuracy of methods.		4			
Please rate the statistical evaluation and quality control.	5				
Please rate the appropriateness of the figures and tables.	5				
Please rate the appropriateness of the references.	5				
Please evaluate the writing style and use of language.	5				
Please judge the overall scientific quality of the manuscript.		4			
Are the methods used worthy of reproduction in greater deal?	No				
Would you be willing to review a revision of this manuscript?	Yes				

Comments to author: Dear authors, thank you for submitting your manuscript "Pit picking versus Limberg flap versus primary open method to treat Pilonidal Sinus Disease – a cohort of 327 consecutive patients" to the journal of ISS. In your article your present your retrospective long-term data on the treatment of PSD with comparing the minimally-invasive technique to open

lay and plastic reconstructive surgeries. Based on your results, and on the high recurrence rates found for pit picking you do not recommend this technique as the standard of care. Your article is well written and sound, and although based on a small, but single-centre collective and its retrospective nature, I would support its publication in ISS. Nevertheless, as the discussion deems little too long, I would recommend shortening this section to an adequate length.

Reviewer 3: Timm Franzke

Date received: 07-Feb-2022
Reviewer recommendation: **Accept in present form**
Reviewer overall scoring: **High**

Assessment Form scores: 5 = High/Yes; 3 = Medium/Adequate; 1 = Low

Is the subject area appropriate for the journal	5				
Does the title clearly reflect the paper's content?	5				
Does the abstract clearly reflect the paper's content		4			
Do the keywords clearly reflect the paper's content?		4			
Does the introduction present the problem clearly?	5				
Are the results/ conclusions justified?			3		
How comprehensive and up-to-date is the subject matter presented?			3		
How adequate is the data presentation?		4			
Are units and terminology used correctly?	5				
Is the number of cases adequate?		4			
Are the experimental methods/ clinical studies adequate?		4			
Is the length appropriate in relation to the content?		4			
Does the reader get new insights from the article?			3		
Please rate the practical significance.			3		
Please rate the accuracy of methods.			3		
Please rate the statistical evaluation and quality control.			3		
Please rate the appropriateness of the figures and tables.		4			
Please rate the appropriateness of the references.		4			
Please evaluate the writing style and use of language.		4			
Please judge the overall scientific quality of the manuscript.		4			
Are the methods used worthy of reproduction in greater deal?	Yes				
Would you be willing to review a revision of this manuscript?	Yes				

Comments to author: Dear Thanks for this study.
Patient selection and, above all, communication with patients seems to be very important. 60% recurrences also mean 40% recurrence-free and this with significantly less pain and without a large wound.
Unfortunately, there is still no information on complications, pain, quality of life, lost work, etc.

Authors' Response to Reviewer Comments

Date received: 15-Feb-2022

Response to reviewer 1

Thank you for this important study.
Since the various minimal invasive treatment options of PSD evolved in rather quick succession over the years, as described in your paper, it is vital for the benefit of our patients to follow up on these techniques in studies like this one.
Of course the number of cases ist rather small over a time period of 9 years, but the data is adequately presented with clear structure and purpose. The data allows and sustains your conclusions.
Just minor changes due to typos should be adressed:
Page 11, Line 57 and following. The text appears to be superscript, also on Page 13 line 7.

Dear Reviewer #1, Thank you very much for taking your time to read and analyse our study. Thanks for your valuable comments and hints that we incorporated into the text in full..

Response to reviewer 2

Dear authors, thank you for submitting your manuscript "Pit picking versus Limberg flap versus primary open method to treat Pilonidal Sinus Disease – a cohort of 327 consecutive patients" to the journal of ISS. In your article your present your retrospective long-term data on the treatment of PSD with comparing the minimally-invasive technique to open lay and plastic reconstructive surgeries. Based on your results, and on the high recurrence rates found for pit picking you do not recommend this technique as the standard of care. Your article is well written and sound, and although based on a small, but single-centre collective and its retrospective nature, I would support its publication in ISS. Nevertheless, as the discussion deems little too long, I would recommend shortening this section to an adequate length.

Dear Reviewer #2, it was a pleasure reading your comments and analysis of our study. We have shortened the discussion according to your comments. Thank you very much for taking your time with our manuscript data.

Response to reviewer 3

Comments to the Author
Thanks for this study.

It is our pleasure, Sir.

Comments by the Editor-in-Chief to Revised Submission – Final Decision

All reviewer comments were addressed by the authors. Reviewer raised only minor points for improvement on the original version. Therefore, I accept the revised manuscript for publication in our journal.
